# *malariaAtlas*: an R interface to global malariometric data hosted by the Malaria Atlas Project

**DOI:** 10.1186/s12936-018-2500-5

**Published:** 2018-10-05

**Authors:** Daniel A. Pfeffer, Timothy C. D. Lucas, Daniel May, Joseph Harris, Jennifer Rozier, Katherine A. Twohig, Ursula Dalrymple, Carlos A. Guerra, Catherine L. Moyes, Mike Thorn, Michele Nguyen, Samir Bhatt, Ewan Cameron, Daniel J. Weiss, Rosalind E. Howes, Katherine E. Battle, Harry S. Gibson, Peter W. Gething

**Affiliations:** 1 Malaria Atlas Project, Big Data Institute, Nuffield Department of Medicine, University of Oxford, Roosevelt Drive, Oxford, OX3 7FY UK; 2 Sanaria Institute for Global Health & Tropical Medicine, Rockville, MD 20850 USA; 3 Department of Infectious Disease Epidemiology, Imperial College London, London, W2 1PG UK

**Keywords:** Malaria, Open-access, Malariometric data, Parasite rate, R package

## Abstract

**Background:**

The Malaria Atlas Project (MAP) has worked to assemble and maintain a global open-access database of spatial malariometric data for over a decade. This data spans various formats and topics, including: geo-located surveys of malaria parasite rate; global administrative boundary shapefiles; and global and regional rasters representing the distribution of malaria and associated illnesses, blood disorders, and intervention coverage. MAP has recently released *malariaAtlas,* an R package providing a direct interface to MAP’s routinely-updated malariometric databases and research outputs.

**Methods and results:**

The current paper reviews the functionality available in *malariaAtlas* and highlights its utility for spatial epidemiological analysis of malaria. *malariaAtlas* enables users to freely download, visualise and analyse global malariometric data within R. Currently available data types include: malaria parasite rate and vector occurrence point data; subnational administrative boundary shapefiles; and a large suite of rasters covering a diverse range of metrics related to malaria research. *malariaAtlas* is here used in two mock analyses to illustrate how this data may be incorporated into a standard R workflow for spatial analysis.

**Conclusions:**

*malariaAtlas* is the first open-access R-interface to malariometric data, providing a new and reproducible means of accessing such data within a freely available and commonly used statistical software environment. In this way, the *malariaAtlas* package aims to contribute to the environment of data-sharing within the malaria research community.

**Electronic supplementary material:**

The online version of this article (10.1186/s12936-018-2500-5) contains supplementary material, which is available to authorized users.

## Background

Since 2005, the Malaria Atlas Project (MAP) has worked to assemble and maintain a global open-access database of spatially explicit malariometric data. This work has been motived by dual aims to both enhance open-access malaria data availability and to provide operationally relevant information for national and international policymakers [1–4]. The availability of this repository of global malariometric data has underpinned numerous studies in the field [5–15]; and continues to support prominent international research such as the Global Burden of Disease study [16, 17] and the World Malaria Report [18–22]. The fundamental need for accurate local information on malaria burden is evident now more than ever, as more countries approach malaria elimination and the challenges of limited funding, insecticide resistance and antimalarial resistance continue to grow [18]. To this end, MAP maintains a routinely updated assembly of national and subnational malariometric data, while developing tools to enable open access to this data for researchers and policymakers worldwide.

The data estate hosted at MAP is one of the largest open-access collections of global malariometric data, both in terms of number of records and geographic coverage. This data spans various formats, topic areas and spatial resolutions, including survey data for precise point locations, administrative-unit level routine surveillance data, and raster grids of spatially continuous modelled predictions (see Table 1). The geo-located survey data specifically encompass: malaria parasite rate (cross-sectional point prevalence), malaria-relevant blood disorder prevalence, intervention coverage, and *Anopheles* vector occurrence. The subnational routine surveillance data covers metrics such as API (annual parasite incidence) and malaria mortality. Finally, the predicted global and regional rasters represent estimates of the distribution of malaria infection and associated disease (e.g. clinical incidence; malaria-attributable fever), malaria-relevant blood disorders, vector occurrence and relative abundance, intervention coverage, and accessibility to cities. This database comprises published data from scientific publications, national surveys (e.g. DHS and MIS [23, 24]), and grey literature produced by national ministries of health and international organizations; as well as unpublished data from researchers and malaria control programmes worldwide. Altogether this represents decades of collaborative work and countless person-hours of on-the-ground data collection.

**Table 1:** Outline of the Malaria Atlas Project open-access data estate and current availability

Data type and format	Open-access availability	
	*malariaAtlas*	Web-tools^a^
*Geo-located point data*		
Malaria parasite rate (PR; for *P. falciparum* and *P. vivax)*	Available now	Available now
Dominant mosquito vectors	Available now	Available now
Malaria-relevant blood disorders	Coming soon	Available now
*Administrative-unit (polygon) level data*		
Administrative boundary shapefiles	Available now	Not currently available
Annual Parasite Incidence (API; for *P. falciparum* and *P. vivax*)	Coming soon	Coming soon
Malaria reproductive number (*P. falciparum)*	Coming soon	Available now
*Global/regional raster grids*		
Predicted malaria infection risk, prevalence, and associated illness	Available now	Available now
Predicted prevalence of malaria-relevant blood disorders	Available now	Available now
Predicted mosquito vector distribution and relative abundance	Available now	Available now
Intervention Coverage (ITNs; IRS; ACT)	Available now	Available now
Global travel time to cities	Available now	Available now

^a^Available at map.ox.ac.uk

Along with a newly released suite of online tools that enable open-access availability to MAP’s databases and associated research outputs (available at http://www.map.ox.ac.uk), MAP has recently released *malariaAtlas,* an R package providing a direct interface to MAP’s open-access databases and research outputs [25–27]. This interface offers three main advantages to traditional data repositories, including: user-defined queries to enable efficient downloading of subsets of large datasets; automatic access to the most up-to-date version of the database including new data and/or database amendments; and transparent and reproducible data access in the form of a few lines of shareable R code. This paper introduces *malariaAtlas,* outlining the available data and functions in the package and illustrating its utility in two reproducible mock analyses.

## Results and discussion

### Data available through *malariaAtlas*

*malariaAtlas* currently enables users to download, visualize and manipulate three types of data: parasite rate (PR) survey data; administrative boundary shapefiles; and a large suite of rasters covering a range of modelled outputs related to malaria research (see Table 1). Georeferenced PR survey data is a core component of MAP’s data estate and a common measure of malaria endemicity [1, 28]. The PR survey points entered into MAP’s database are screened for robust sampling methods and geographic specificity to ensure they provide representative parasite species-specific information on the local prevalence of malaria infection. This database includes 73,326 survey points as of July 2018 (64,685 *Plasmodium falciparum*; 14,412 *Plasmodium vivax*), covering the period 1975–2017. In addition to georeferenced data on malaria endemicity, up-to-date and topologically correct shapefiles of a region’s administrative boundaries are fundamental to visualizing, interpreting and analysing spatial epidemiological data. As such, MAP maintains a collated set of subnational administrative boundary shapefiles assembled from various publicly available sources (see [29]). MAP also makes a large number of raster grids publicly available, representing the major outputs of MAP’s spatiotemporal epidemiological research. At the time of writing, 86 raster surfaces were available to download using *malariaAtlas*. These cover a variety of relevant metrics, such as predicted malaria parasite prevalence, clinical incidence and malaria-attributable fever [8, 30–32]; prevalence of malaria-related human blood disorders [33–35]; predicted risk of zoonotic *Plasmodium knowlesi* infection [36]; predicted mosquito vector distribution and relative abundance [37–40]; coverage of insecticide-treated bed nets (ITNs), indoor residual spraying (IRS) and artemisinin-based combination therapy (ACT) [8]; and travel time to cities [41]. By providing an R-interface to MAP’s hosted survey data, shapefiles and rasters, *malariaAtlas* enhances direct and reproducible access to this data source.

### Downloading and visualizing data with *malariaAtlas*

Using *malariaAtlas* to download and visualize data from MAP in R is achieved through four main classes of functions as outlined in Table 2. These include: ‘list’ functions that allow the user to see how much data is available for a given data type; ‘get’ functions for data downloads; ‘*autoplot’* methods that enable quick visualisation of downloaded data using functions from the *ggplot2* package [42]; and a number of utility functions that enable common manipulations of downloaded data (see Table 2).

**Table 2:** Outline of *malariaAtlas* functions

Category	Function name	Purpose	Data type	R object class
‘List’ available data	*listData*	Wrapper for below functions, returning a *data.frame* outlining data availability	–	*data.frame*
	*listPoints*	Return a *data.frame* listing countries with parasite rate survey points	Point data	*data.frame*
	*listShp*	Return a *data.frame* listing administrative units with shapefiles available to download	Shapefile	*data.frame*
	*listRaster*	Return a *data.frame* listing rasters available to download	Raster	*data.frame*
‘Get’ available data	*getPR*	Download parasite rate survey data for specified location(s) and species	Point data	*data.frame;* *pr.points* ^*a*^
	*getShp*	Download shapefiles for specified location(s) and administrative level(s)	Shapefile	*SpatialPolygon(s);* *data.frame; mapShp* ^a^
	*getRaster*	Download specified rasters for specified location(s) and year(s)	Raster	*RasterLayer; RasterBrick; RasterStack; data.frame;* *mapRaster* ^*a*^
‘*Autoplot’* downloaded data	*autoplot.pr.points*	Quickly visualise parasite rate survey locations and raw PR values for data downloaded using *malariaAtlas*	Point data	*gg*
	*autoplot.mapShp*	Quickly visualise shapefiles downloaded using *malariaAtlas*	Shapefile	*gg*
	*autoplot.mapRaster*	Quickly visualise rasters downloaded using *malariaAtlas*	Raster	*gg/list*
Utility functions	*extractRaster*	Extract values from specified rasters at specified point locations (lat/long)	Point data	*data.frame*
	*convertPrevalence* ^b^	Convert parasite rate from a given age-range to another	Prevalence	*numeric*
	*as.mapShp*	Convert *SpatialPolygon* or *SpatialPolygons* objects to *mapShp*^*a*^ objects	Shapefile	*mapShp*^*a*^; *data.frame*
	*as.mapRaster*	Convert objects of *RasterLayer*; *RasterBrick*; *RasterStack* classes or a *list* of *RasterBrick*/*RasterStacks* to *mapRaster*^*a*^ objects	Raster	*mapRaster*^*a*^; *data.frame*

^*a*^ *malariaAtlas* specific object class defined for purposes of quick visualisation using *autoplot* (*pr.points*; *mapShp*; and *mapRaster*) or in-built optional conversion of Spatial* classes to *data.frame* formats (*mapShp*; *mapRaster*)

^b^ See the *ageStand* R package on GitHub [43] or *malariaAtlas* help files for additional information on *convertPrevalence*

**Fig. 1:**
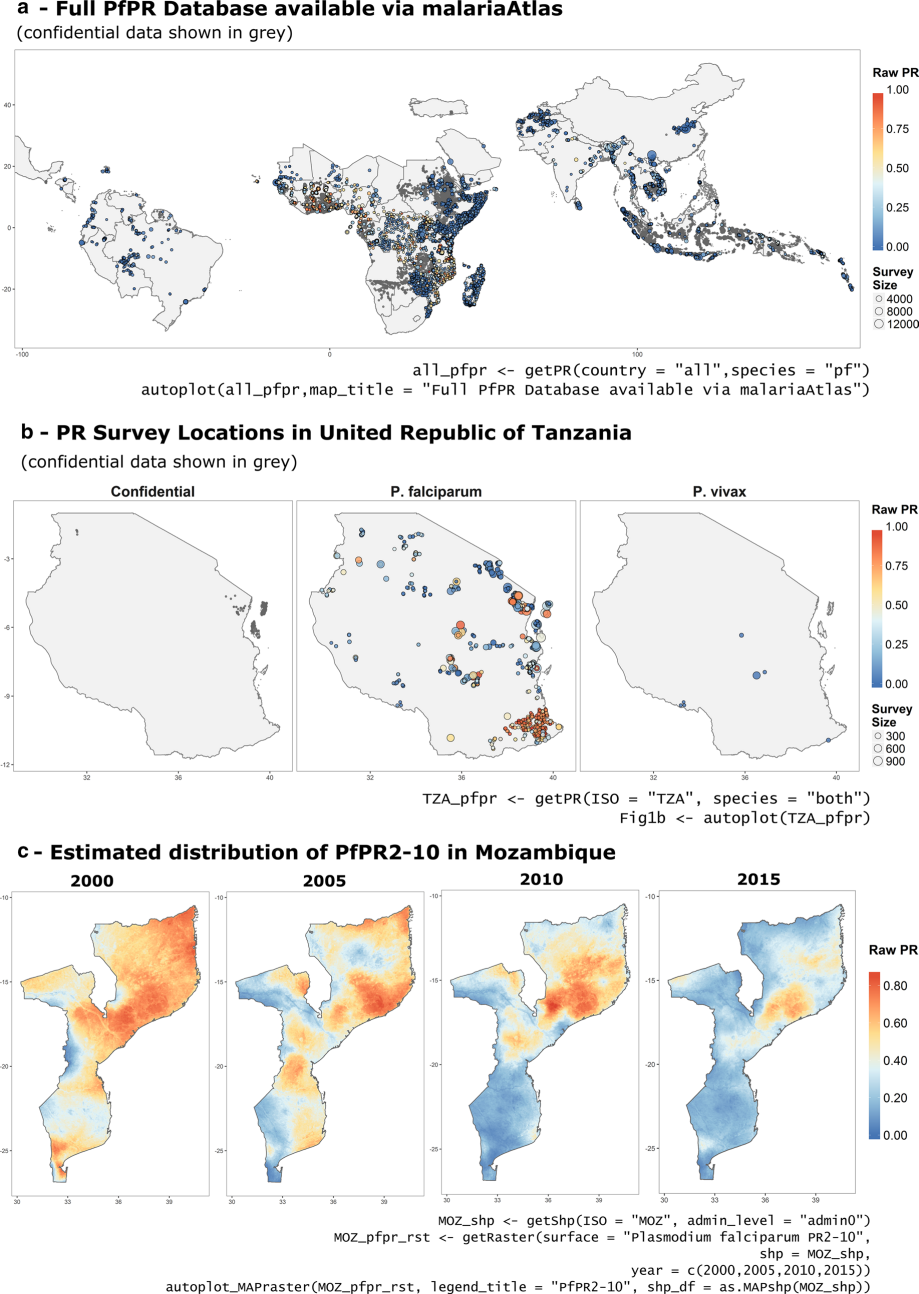
Using *malariaAtlas* to download and visualise geolocated parasite rate data and modelled raster data. **a** *malariaAtlas*-derived map of the full *Pf*PR database available to download using *getPR*. Points are coloured according to PR value and sized according to sample size. Grey points illustrate confidential data. **b** Map of all PR points from The United Republic of Tanzania hosted by MAP for both *Plasmodium falciparum* and *Plasmodium vivax*. **c** Rasters of estimated spatial distribution of *Pf*PR in Mozambique in 2000, 2005, 2010 and 2015 from Bhatt et al. [8]. For all panels, the *malariaAtlas* R code used to download and visualise the relevant data is included below the map

Within *malariaAtlas*, the functions *listPoints*, *getPR* and *autoplot.pr.points* provide a quick and simple way of downloading and visualising publicly available PR survey data hosted by MAP. *listPoints* returns a *data.frame* outlining the countries for which parasite rate survey data is available in MAPs database. *getPR* returns a *data.frame* of geo-located PR point data including: number of individuals examined; number of positive diagnoses by species; age range of the sample population; sampling date and location information; diagnostic method(s) used; and source citation. Arguments are included to enable queries based on location (Continent; Country Name; 3 letter ISO code; or spatial extent) and species (either *P. falciparum* or *P. vivax*). The returned data has the additional class ‘*pr.points’* which enables quick visualization of downloaded points using *autoplot*. A subset of the PR survey points in MAP’s database remain confidential, in accordance with the respective data-use agreements under which they have been shared. For these confidential data points, MAP has either limited or no permission to share measured PR values and/or geo-location data, however citations to the original data source are provided for all downloaded points. Accordingly, data-sharing restrictions for any given point are provided in the ‘permissions_info’ column of a downloaded *pr.points data.frame*. Figure 1 illustrates the use of *malariaAtlas* to download and visualise PR survey points, including maps of (a) the full database of available *P. falciparum* PR points at the time of publication (Fig. 1a, b) all PR survey points hosted by MAP from Tanzania (Fig. 1b).

Analogous to the functions described above, *listShp*, *getShp* and *autoplot.mapShp* allow users to download and visualise the set of shapefiles collated by MAP (see Table 2). *listShp* returns a *data.frame* indicating all administrative regions covered by these shapefiles along with their administrative level and corresponding parent administrative unit. *getShp* returns either a *SpatialPolygons* object or *mapShp* object (as chosen by the user) containing polygons at either ADMIN0 (national) or ADMIN1 (state; province) levels for any given country; and down to ADMIN3 level for some malaria-endemic countries. Quick visualisation of *mapShp* objects is possible through an *autoplot* method.

Rasters are the final datatype available to download and visualise using *malariaAtlas* via the functions: *listRaster*, *getRaster* and *autoplot*.*mapRaster*. *listRaster* returns a *data.frame* that serves as a catalogue of rasters available to download using *getRaster,* mirroring the catalogue of rasters available on MAP’s online interactive explorer tool (map.ox.ac.uk/explorer). This *data.frame* includes columns that provide descriptive metadata including an abstract outlining raster content, a citation to the original publication associated with a given raster, and the time period covered for time-varying raster datasets. *getRaster* provides the means to download one or more raster layers at a time, queried by location (using either an input *SpatialPolygon* shapefile or a user-defined extent (xmin, xmax, ymin, ymax)), and year (for temporally dynamic raster datasets). The data is returned as a Raster* object: a *RasterLayer* for a single raster; a *RasterBrick* for two or more rasters of the same extent/resolution; or a *list* of Raster* objects for two or more rasters of differing extents/resolutions. Downloaded rasters represent the mean predicted value from various geostatistical models. For further information on specific modelling approaches and/or associated uncertainty of predicted values users are encouraged to consult the associated publication (citation information available via *listRaster*) or to contact MAP directly. The utility function *as.mapRaster* converts any object downloaded using *getRaster* into a *mapRaster* object (long-format *data.frame* with columns x, y, z (longitude, latitude, value) and raster_name) enabling tabular manipulation and *ggplot*-friendly visualisation. Quick visualization of *mapRaster* objects is provided via included *autoplot* methods. Figure 1c illustrates example code used to download and quickly visualise a raster for a given shapefile extent via *malariaAtlas*.

### Data manipulation and utility functions

Three additional utility functions have been added to provide an easy means to perform common data manipulations. *extractRaster* allows users to download values from MAP rasters at specific point locations supplied in a user-specified set of coordinates (see *malariaAtlas* Vignette; [27]). This enables users to input a list of locations (latitude, longitude) and get back the associated raster value (e.g. malaria prevalence) for each location. *as.mapShp* and *as.mapRaster* provide a means of converting between Spatial* class objects (for polygon data) or Raster* class objects (for raster data) to the *malariaAtlas data.frame*-based object classes *mapShp* and *mapRaster* respectively. This permits tabular manipulation and *ggplot*-friendly plotting through provided *autoplot* methods. *convertPrevalence* is an additional utility function that provides a principled approach to age-standardization of malaria prevalence data [43], based on models defined by Smith et al. [28] for *P. falciparum* and Gething et al. [30] for *P. vivax*. Altogether, the above functions provide a simple means of downloading, visualising and manipulating spatial malariometric data. The flexibility of R as a statistical software platform and the wealth of existing R packages enable users to easily extend their analysis beyond these functions and integrate *malariaAtlas* into more complex analytical workflows.

### *Zoon* modules

To further aid the dissemination and use of these data, *malariaAtlas* modules were developed for the species distribution modelling software *zoon *[44]. *Zoon* provides a modular framework for species distribution modelling, allowing users to collect and model data in a simple pipeline. Species distribution modelling is a subfield of ecology in which the spatial distribution of an organism is estimated from known presence and absence (if available) locations. There are strong parallels between species distribution modelling and parasite rate mapping as both use binomial data to estimate a spatial probability surface; in species distribution modelling this surface is the probability of species occurrence while in parasite rate mapping the surface is probability of infection. Two *zoon* modules have been added (‘*malariaAtlas_PR*’ and ‘*malariaAtlas_covariates*’*)* allowing parasite rate surveys to be used as response data and raster data to be used as covariates within a *zoon* workflow. The parasite rate data offers a useful benchmark dataset for testing new methods. However, state-of-the-art models of malaria prevalence (e.g. [8]) are currently beyond the scope of *zoon,* and as such *zoon* is not expected to be directly used for risk mapping and/or policymaking.

### Mock analysis 1: predicting the spatial distribution of *Plasmodium vivax* using *malariaAtlas*-derived response and covariate data

**Fig. 2:**
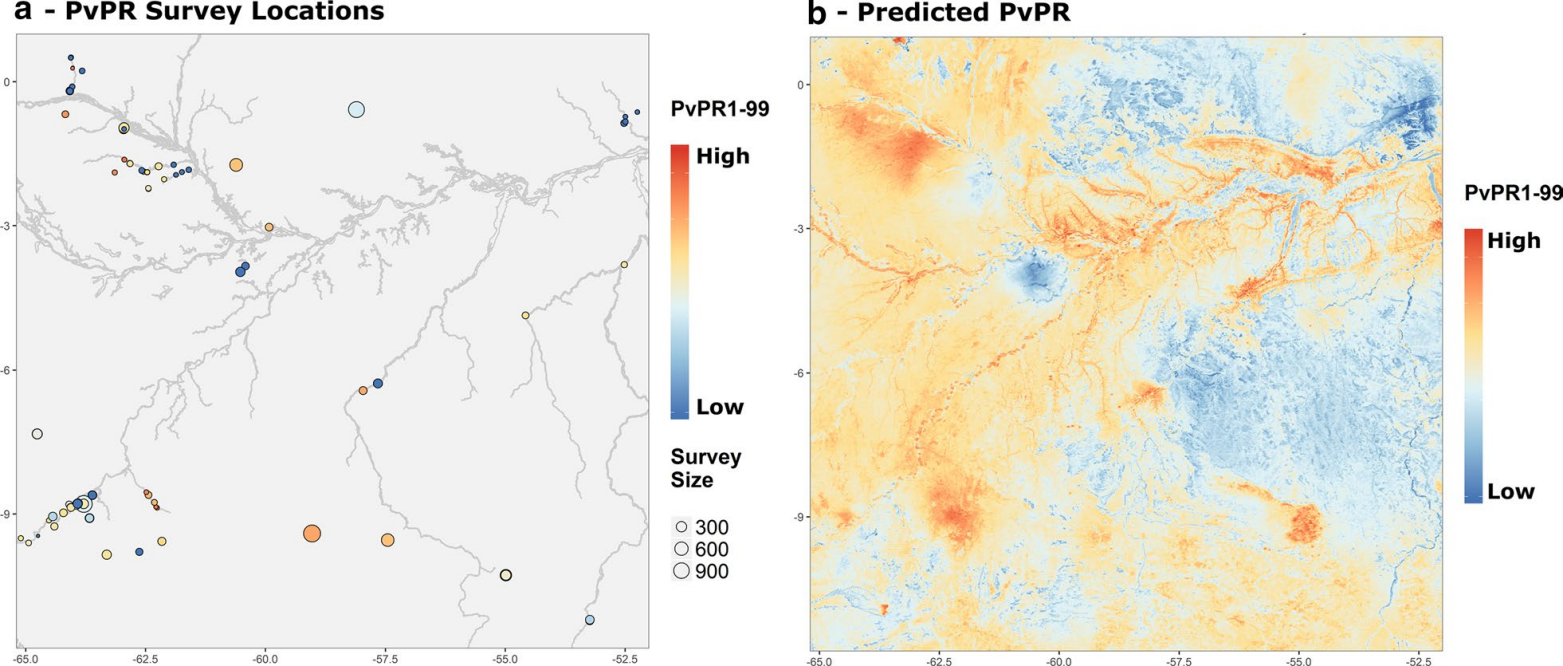
Predicting the spatial distribution of *Plasmodium vivax* using *malariaAtlas*-derived response and covariate data. **a** Map illustrating locations of age-standardised *Pv*PR survey points within the study area as used for response data in mock analysis 1. River locations were downloaded from the Global Lakes and Wetlands Database [52]. **b** Predicted *Plasmodium vivax* parasite rate within the study area. Predictions are derived from a Bayesian geostatistical model using data in panel **a** and environmental covariates including night-time temperature, elevation and rainfall. Both maps were produced using *malariaAtlas*’ *autoplot* methods and *ggplot2* [42]. Absolute values were removed from the colour scales to reflect the purely illustrative nature of this analysis

The first mock analysis illustrates the use of *malariaAtlas* to download response and covariate data for use in spatial epidemiological analysis. *P. vivax* parasite rate (*Pv*PR) survey points and covariate raster data were downloaded using *malariaAtlas* (see Box 1) and used to fit a Bayesian geostatistical model of malaria risk (see full example code in Additional file 1). For illustrative purposes, an arbitrary spatial extent was chosen for this analysis. All *Pv*PR points in the study area were downloaded using *getPR,* and then subsetted to only publicly available data for analysis. *convertPrevalence* was used to standardize values to all-ages *Pv*PR (see Fig. 2a; Box 1). The R-INLA package [45, 46] was used to fit a Bayesian geostatistical model with a binomial likelihood to these data. Covariate data included rasters of environmental factors (night-time land surface temperature [47]; log elevation [48]; rainfall [49]) and log travel time to the nearest city (downloaded using *getRaster* as in Box 1, hereafter referred to as ‘human accessibility’; [41]). These fixed effects were given minimally informative (INLA default) priors.
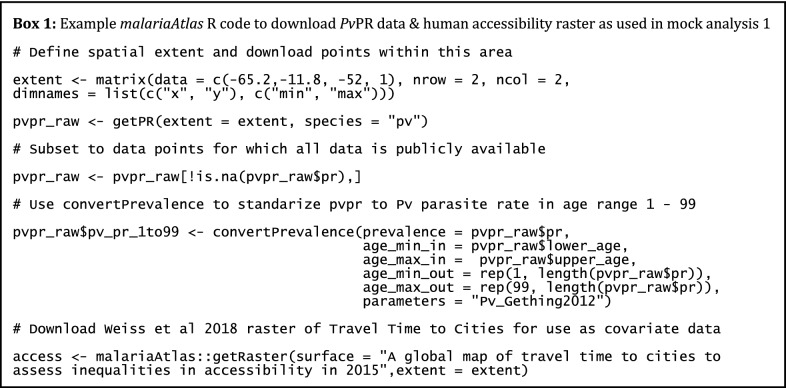

The spatial autocorrelation in the data was modelled using a continuous, spatial Gaussian random field with a Matern covariance function [45]. The hyperparameters of the random field were given Penalised Complexity (PC) priors, which by design prefer a simpler model with a smoother random field [50]. The hyperparameters of the random field are the range (the distance within which the correlation of the field is essentially zero) and the standard deviation (the amount the field can vary). For the current model, the priors on these values were parameterised by setting the probability that the range of the field was smaller than an extreme minimum value (2 decimal degrees) as 0.01 and the probability that the standard deviation of the field was greater than an extreme maximum value (2.7) as 0.01. A random field with a standard deviation of 2.7 would be able to explain all the residual variance from a previously fitted logistic regression. The above prior was thus defined such that this undesirable level of overfitting was unlikely.

The fitted model was used to predict *Pv*PR across the spatial extent of the study area (see Fig. 2b). Within this model, night-time temperature and elevation were significant predictors of *Pv*PR (estimated coefficients (95% CI) of − 0.98 (− 1.70 to − 0.30) and − 1.43 (− 2.69 to − 0.38) respectively), while human accessibility did not significantly predict *Pv*PR (− 0.16 (− 0.44 to 0.16)). Overall interpretation of these results is limited due to its small sample size and arbitrary spatial extent. Nevertheless, this mock analysis illustrates the use of *malariaAtlas* to download spatial malariometric response and covariate data for incorporation into further analysis.

### Mock analysis 2: testing a new modelling approach using in-built *malariaAtlaszoon* modules

**Fig. 3:**
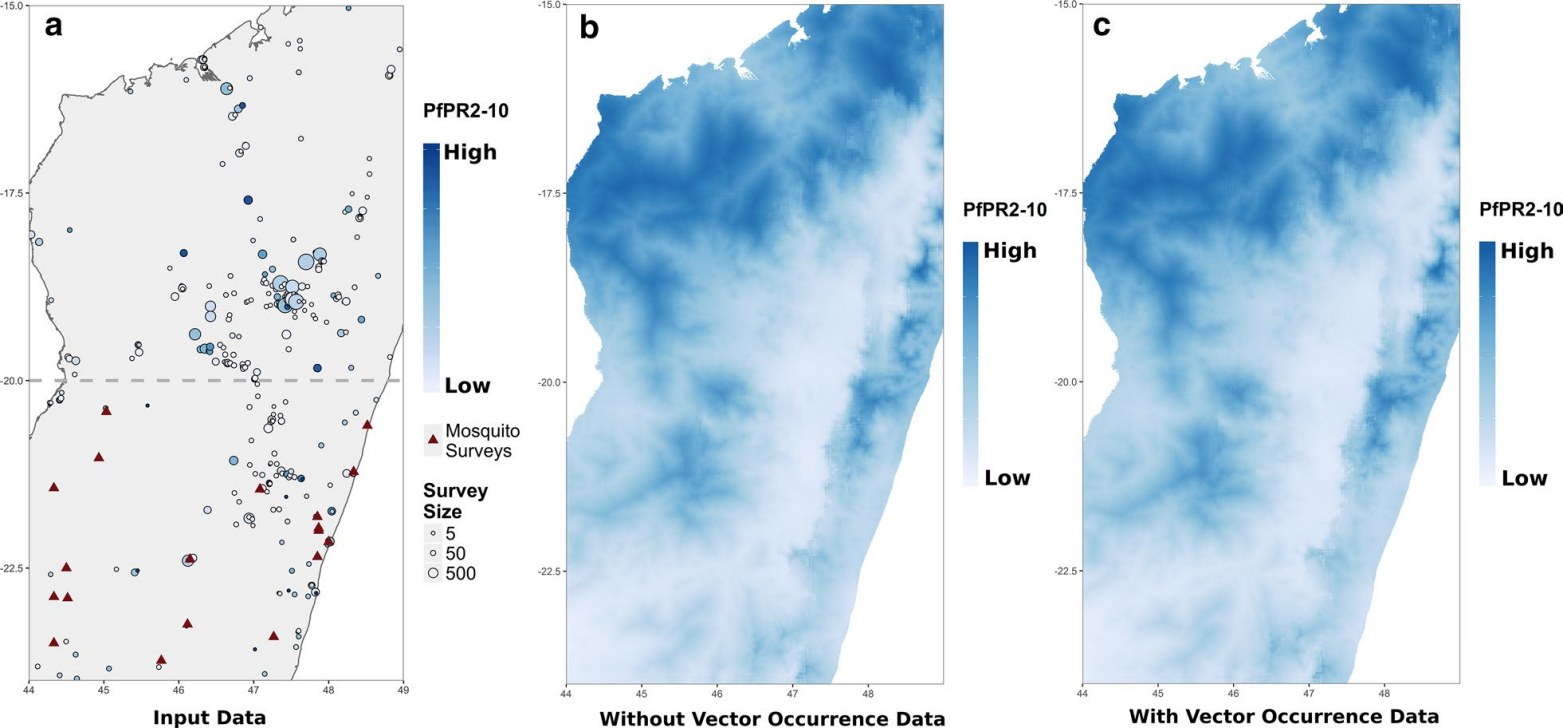
Including mosquito occurrence data alongside PR survey data in models of *Plasmodium falciparum* parasite rate. **a** Map of geolocated input data, PR points (coloured circles) were obtained from MAP using the *malariaAtlas_PR zoon* module; mosquito presence data (red triangles) were obtained from GBIF using the *SpOcc zoon* module [44, 51, 53]. **b**, **c** Predicted *Plasmodium falciparum* parasite rate from logistic regression models using either PR data only (**b**) or PR data and mosquito occurrence data (**c**). Maps were produced using *malariaAtlas*’ *autoplot* methods and *ggplot2* [42]. Absolute values were removed from the colour scales to reflect the purely illustrative nature of this analysis


The second mock analysis demonstrates how *malariaAtlas* can be used to access malariometric data within a *zoon* workflow [44]. As an illustrative example, this analysis investigates whether including mosquito occurrence data can improve predictive models of *Pf*PR, using data from a second arbitrary study area (bounded by latitudes of − 24 and − 15 and longitudes of 44 and 49). A simple spatial validation scheme was implemented, using *Pf*PR data from north of latitude − 20 (28,921 individuals from 208 locations) as a hold-out validation data set. Logistic regression models were fitted to two datasets and their predictive performance was compared. The first data set was simply the *Pf*PR data from 116 locations and 8546 individuals south of latitude − 20. The second dataset was comprised of the same *Pf*PR data with the addition of known occurrence locations of *Anopheles arabiensis* and *Anopheles gambiae* collected from the Global Biodiversity Information Facility [51], treating each vector occurrence location as equivalent to a single positive case of *P. falciparum* (total 147 locations and 8592 individuals/mosquitoes; see Fig. 3a). For covariates, WorldClim layers 1, 4, 12 and 15 (mean and within-year variation of temperature and precipitation [49]) as well as human accessibility [41] were used. *Pf*PR data and human accessibility rasters were downloaded using *malariaAtlas zoon* modules (see Box 2). Model performance was compared using the AUC (Area Under the Curve) model evaluation criterion which assessed the ability of each model to correctly assign an infected/non-infected status to individuals in the hold-out set.

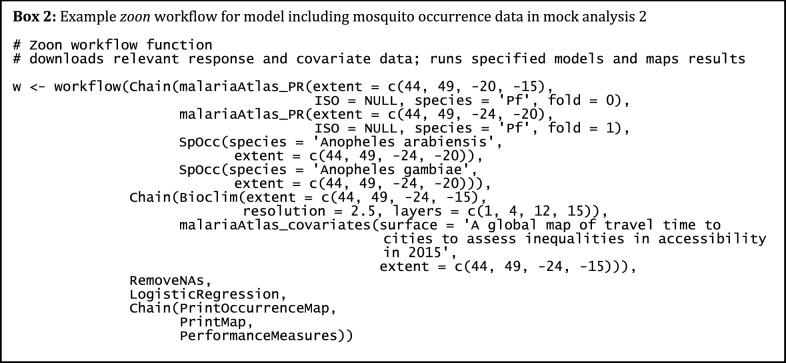


Including mosquito occurrence data very marginally improved predictive performance. AUC was 0.577 without mosquito occurrence data and 0.578 with the addition of mosquito data. Maps created using both models are shown in Fig. 3b, c showing almost identical outcomes. It is worth noting that the difference in model performance has no practical relevance. However, this serves as an illustrative example of how *malariaAtlas* data can be used within *zoon* to test new methods. Larger scale comparisons, and a less naive approach to incorporating mosquito data, would be needed to truly examine whether this method has analytical merit.

## Conclusions

*malariaAtlas* is the first open-access R-interface to malariometric data, providing a new and reproducible means of accessing this data within a freely available and commonly used statistical software environment. As such, by using *malariaAtlas*, any individual with internet access can directly download, visualise and analyse data from the Malaria Atlas Project. Furthermore, this package is designed to fit into existing research workflows, enabling importation of multiple data-types in a few simple lines of code, as illustrated in the mock analyses above. As the MAP data estate continues to grow, *malariaAtlas* will offer an up-to-date interface to the most recent malariometric data. Future updates will seek to provide access to additional data-types (e.g. publicly reported routine surveillance data; site-level geolocated survey data of other types such as prevalence data for glucose-6-phosphate dehydrogenase deficiency and the Duffy negative blood group; and new raster datasets such as modelled resistance to the insecticides used in malaria control). Future updates will also include the option for date-specific data queries (e.g. ‘download data as at 01/04/2018’), enabling truly reproducible data download irrespective of potential amendments to the source database. *malariaAtlas* rests upon decades of valuable collaboration and data-sharing within the malaria research community. By providing a new means of open-access to malariometric data it is hoped that this package both contributes to this environment of open data-sharing and also provides a valuable tool to malaria researchers worldwide.

## Additional file

**Additional file 1.** Illustrative R code used to conduct the Mock Analyses in this paper.
